# Mode of HIV exposure and excess burden of neurocognitive impairment in people living with HIV: a protocol for systematic review and meta-analysis of controlled studies

**DOI:** 10.1186/s13643-023-02371-6

**Published:** 2023-11-16

**Authors:** Astri Parawita Ayu, Arie Rahadi, Kevin Kristian, Tara Puspitarini Sani, Aditya Putra, Glenardi Halim, Ghea Mangkuliguna, Theresia Puspoarum Kusumoputri, Yuda Turana

**Affiliations:** 1 Department of Psychiatry and Behavioural Sciences, School of Medicine and Health Sciences, Atma Jaya Catholic University of Indonesia, North Jakarta, 14440 Indonesia; 2 HIV AIDS Research Center (ARC) – University Center of Excellence in Health Policy and Social Innovation, Atma Jaya Catholic University of Indonesia, South Jakarta, 12930 Indonesia; 3 Department of Public Health and Nutrition, School of Medicine and Health Sciences, Atma Jaya Catholic University of Indonesia, North Jakarta, 14440 Indonesia; 4 Alzheimer Indonesia, South Jakarta, 12930 Indonesia; 5 Department of Neurology, School of Medicine and Health Sciences, Atma Jaya Catholic University of Indonesia, North Jakarta, 14440 Indonesia; 6 Atma Jaya Neuroscience and Cognitive Centre, Atma Jaya Hospital, North Jakarta, 14440 Indonesia

## Abstract

**Background:**

Chronic HIV infection significantly elevates the risk of brain pathology, precipitating neurocognitive impairment (NCI) among people living with HIV (PLWH). The diagnosis of NCI in PLWH hinges on evaluating deviations in neuropsychological test performance in comparison to HIV-seronegative normative controls. However, the adverse psychosocial conditions experienced by PLWH can also result in reduced test performance, potentially confounding the accurate NCI attribution to HIV infection. This planned systematic review aims to investigate potential disparities in the excess burden of NCI among PLWH in two groups of studies: (a) studies enrolling controls who shared a similar mode of HIV exposure (MoHE) with the PLWH participants (MoHE-adjusted) and (b) studies enrolling normative controls or controls without undefined MoHE (MoHE-naive).

**Methods:**

We will systematically search five electronic databases (MEDLINE, Embase, PsycINFO, Web of Science, ProQuest) and registries (OpenGrey, ClinicalTrials.gov, ISRCTN registry). Studies reporting NCI in PLWH and HIV-seronegative controls with cross-sectional or baseline measurements, published from January 2007 to September 2023, will be included. To be classified as MoHE adjusted, a study must evidence ≥ 90% enrolment of both PLWH and their seronegative controls from the same MoHE group (e.g. men who have sex with men, people who use drugs or alcohol). Reports of test performance scores will be transformed into NCI proportions using simulated score distributions, applying a global deficit score cut-off ≥ 0.5 to estimate NCI cases.

The Newcastle–Ottawa scale adapted to the purpose of the review will be used to appraise study quality. Random-effects meta-analysis will be used to pool the excess burden of NCI in prevalence ratios and test the difference between MoHE-adjusted and MoHE-naive studies. Furthermore, subgroup analyses and meta-regression will be undertaken across categorical study-level covariates (e.g. study locations, NCI diagnostic criteria) and continuous/ordinal covariates (nadir CD4, number of neurocognitive domains assessed), respectively.

**Discussion:**

This systematic review will contribute towards a greater appreciation of the unique psychosocial conditions of PLWH that are missing from the current case definition of HIV-associated neurocognitive disorder. The findings will additionally highlight possible disparities in the distribution of the excess burden of NCI by MoHE groups, thereby guiding the prioritization of mitigation efforts.

**Systematic review registration:**

PROSPERO CRD42021271358

**Supplementary Information:**

The online version contains supplementary material available at 10.1186/s13643-023-02371-6.

## Background

The combination antiretroviral treatment (cART) has substantially reduced mortality among people living with HIV (PLWH) worldwide [1–4]. These gains in life expectancy, however, have been characterized by distinctive manifestations of morbidities resulting from the interplay of chronic HIV infection with the ageing process [5–7]. In neurocognitive health, the advent of cART has shifted the burden of HIV-associated neurocognitive disorders (HAND) from the severe form of neurocognitive impairment known as AIDS dementia complex to a preponderance of milder impairments either with or without accompanying symptoms [8]. Two recent meta-analyses have estimated the global prevalence of HAND to exceed 40% among PLWH on the basis of the widely used Frascati diagnostic criteria [8]. Approximately, 90% of this burden was estimated to be asymptomatic or exhibit mild impairments, while the remaining portion is attributed to HIV-associated dementia [9, 10]. While emphasizing the pressing need for effective therapies, this seemingly elevated burden of neurocognitive disorders in PLWH lacks context in terms of attributing the risk of neurocognitive impairment (NCI) to HIV infection due to the absence of a comparison with burden estimates in the HIV-uninfected population using the same diagnostic criteria.

The putative risk factors of HAND are biologically multifactorial and interlinked with the psychosocial determinants that shape the lives of PLWH [11]. Several key contributors to neurocognitive decline, such as metabolic or cardiovascular diseases and gene variants, become more prominent with ageing in the general population and more so in PLWH [12, 13]. The intrinsic capacity to slow or reverse this trajectory of decline encapsulated in a neuropsychological construct of ‘cognitive reserve’—of which duration of formal education is a key measure [14], also seems to be disproportionately lower in PLWH [15]. It is acknowledged that the pathogenesis of HAND starts with the replication of HIV proteins in the central nervous system due to compromised immunity during sustained viremia [16]. This process among others triggers the activation of microglia and their interaction with chemokine receptors to stimulate chronic neuroinflammation and neuronal damage [16, 17]. Given this immune-mediated biomechanism underpinning HAND, the extent of decline in cognitive functioning can be effectively gauged by nadir CD4 counts [18].

A growing number of reports have begun to examine the psychosocial aetiology of NCI [19–21]. PLWH and those at risk of the infection share risk behaviours, or a mode of HIV exposure (MoHE), which carry high social stigma due to illegality (e.g. drug use) or nonconformance to societal norms in personal choice of lifestyle (e.g. homosexuality, non-monogamy). This stigma and marginalization can adversely impact cognitive health through induced depression and anxiety [22], by limiting social contacts with implications for brain structural integrity [23], being a chronic stressor that increases levels of neuroinflammatory cytokines [24, 25], and by internalization of negative beliefs and stereotypical expectations that may manifest to bias neurocognitive performance in a testing situation [26].

Thus, the psychosocial aetiology theory of NCI recognizes that individuals with a shared MoHE also likely share a baseline neurocognitive performance that can deviate from that of ‘healthy controls’ or the ‘normative population’, irrespective of HIV serostatus [11]. As the diagnosis of HAND involves evaluating deviations in performance compared to the HIV-seronegative group, studies enrolling healthy controls may overstate the rates of NCI attributable to HIV-related brain pathology due to confounding from adverse psychosocial effects linked to the MoHE in PLWH participants. A common strategy to address this confounding effect is to apply stringent exclusion criteria to enrol only exceptional participants with no comorbidities or undesirable psychosocial attributes (e.g. low education, drug use) that could lead to reduced test performance during a neuropsychological evaluation. Nevertheless, this strategy provides limited value in studying a representative population of PLWH, given the well-documented presence of comorbidities and adverse psychosocial conditions [11].

In light of this context, we propose to systematically review published studies of NCI in PLWH. As previously mentioned, prior reviews have quantified the burden of HAND in absolute terms, with likely overestimations. To address this issue, examining variations in how the normative population is defined across studies could reveal potential differences in NCI burden estimates. This protocol proposes a measurement of excess burden of NCI in PLWH, calculated from the relative difference in outcome rates between serostatus groups. By proxying the distribution of intergroup confounding to whether or not seronegative controls share a MoHE with PLWH participants, we hypothesize that the excess NCI burden will diverge between studies where both serostatus groups share a MoHE and studies enrolling healthy controls in the seronegative group, who we postulate to have little exposure to adverse psychosocial conditions compared to PLWH. This protocol has been designed to test this hypothesis and answer the following main questions: (a) What are the estimates of excess burden of NCI in PLWH in studies enrolling participants from the same MoHE across both serostatus groups and in studies enrolling healthy controls? (b) Do the estimates of excess burden differ for MoHE studies in overall and between groups of MoHE? and (c) To what extent do variations in study-level demographics, clinical and methodological characteristics, and MoHE groups modify these estimates?

## Methods

### Protocol registration

In developing this protocol, the guidelines of the Preferred Reporting Items for Systematic reviews and Meta-analysis Protocols (PRISMA-P) were followed (Additional file 1) [27]. A summary of the protocol was registered on the International Prospective Register of Systematic Reviews (PROSPERO) (CRD42021271358). The conduct of the systematic review and meta-analysis will adhere to the Meta-analyses Of Observational Studies in Epidemiology (MOOSE) guideline [28].

### Search strategy and terms

Searches for relevant reports will be conducted across the following databases: MEDLINE (Ovid), Embase (Ovid), PsycINFO (Ovid), Web of Science, and ProQuest. Additionally, we will expand our search to include a grey literature database (OpenGrey) and trial registries (ClinicalTrials.gov, International Standard Randomised Controlled Trial Number [ISRCTN] registry) for potential reports or ongoing or completed studies otherwise not identified in the five main databases. Furthermore, we will complement database searches with hand-searching from the reference list of eligible studies. A sensitive search strategy will be employed with an emphasis on identifying reports of NCI in PLWH. Additional file 2 provides sample search terms we developed for the Ovid databases. We will limit our search to reports published from 1 January 2007 up to 30 September 2023. The start date corresponds to the year of the publication of the revised nosology for HAND which recognizes asymptomatic manifestation of NCI in PLWH [8]. We extended the end date from initially on 31 May 2021 to ensure that the review remains current and up to date.

### Eligibility criteria

Cross-sectional or observational cohort studies reporting counts or proportions diagnosed with NCI, or alternatively neuropsychological test performance scores as the basis for estimating such proportions, in both PLWH and HIV-seronegative participants will be included in the review. Reports of NCI proportions must encompass a full spectrum of NCI, including the following: asymptomatic neurocognitive impairment, HIV-associated mild neurocognitive impairment, and HIV-associated dementia [8]. We will exclude reports on paediatric populations (age < 12 years), reports with < 50 participants across HIV serostatus groups, assessments of < 2 of the recommended six neurocognitive domains [8], conference materials, other study designs (i.e. randomized trials or designs with predetermined selection of outcomes in either serostatus group), and studies enrolling historical controls.

### Study selection

Records will be managed and stored using EndNote. After the removal of duplicates, editorials, and conference materials, two reviewers (A. P., G. H.) will independently screen the titles and abstracts of the remaining records, supervised and adjudicated by a third reviewer (A. R.) in cases of disagreement. We will contact study authors for reports to which we have no full access. Subsequently, two reviewers (A. P. A., A. R.) will assess the eligibility of each report in full text. The study selection process will be summarized in a PRISMA flow diagram [28].

### Outcomes and measures

The primary outcome under investigation is the diagnosis of NCI by study definition. We anticipate a diversity of diagnostic criteria being used across the included studies, which may encompass the following: (a) the Frascati criteria (≥ 1 SD below the mean of normative test scores in ≥ 2 neurocognitive domains [8]); (b) the Gisslén criteria (≥ 1.5 SD below the mean of normative test scores [29]); (c) the global deficit score (the average of reclassified *T* scores on a 0 to 5 scale for all assessed neurocognitive domains, with a cut-off score exceeding 0.5 to delineate impairment [30]); (d) clinical rating scale (reclassified *T* scores on a 1 to 9 scale, with scores exceeding 5 in ≥ 2 domains to evidence impairment [30]); (e) multivariate normative comparisons (use of multivariate statistics to construct and compare profiles of test scores [31]); and (f) various cut-off criteria of instrument-specific screening analogues (e.g. Cogstate [32], International HIV Dementia Scale [33], Mini Mental State Examination [34], or Montreal Cognitive Assessment [35]).

The excess burden of NCI will be measured in prevalence ratios (PR), which is the ratio of NCI proportions in seropositive participants to seronegative participants. We will utilize the frequency of cases and non-cases or the reported proportions for each serostatus group to derive crude PR estimates or regression coefficients for adjusted PR estimates. Measures reported in odds ratio (OR) will be converted to PRs by dividing the OR by one minus the product of multiplying the NCI proportion in the seronegative group with the difference in the OR from unity [36].

### Data extraction and management

We will classify reports that meet the eligibility criteria into two groups: (a) ‘MoHE-adjusted controls’, whereby ≥ 90% of both HIV-seropositive and HIV-seronegative participants share a single MoHE group, or (b) ‘MoHE-naive controls’, whereby the MoHE is either undefined or had a distribution of < 90% in either serostatus group. We will also record information on the following:

- ▪ Participant eligibility (e.g. exclusion due to pre-existing central nervous system disorders, major depression, or substance dependence)
- ▪ Demographics (age, sex, years of education)
- ▪ Comorbid conditions applicable to all participants (e.g. hepatitis C, depression)
- ▪ MoHE groups (e.g. men who have sex with men, people who use drugs or alcohol, people exposed to perinatal HIV infection) for reports of MoHE-adjusted controls
- ▪ HIV clinical characteristics (nadir CD4 counts, the proportions of HIV-seropositive participants on cART)
- ▪ Neurocognitive evaluation (i.e. evaluation test instrument, number of neurocognitive domains assessed, diagnostic criteria for NCI)
- ▪ The frequency of NCI or neuropsychological test scores by HIV serostatus
- ▪ Analytical adjustments (i.e. none, demographics, and/or comorbidities)

Two reviewers (A. P., A. R.) will independently extract these study-level characteristics and outcomes onto a pre-piloted form (Additional file 3), which will then be exported to a spreadsheet to facilitate analysis. The reviewers had been trained to use the extraction form. Disagreements will be resolved through discussion involving expert clinicians in neurocognition and HIV (A. P. A., Y. T.). For education reported in ordinal levels, we will assign a standard duration for each level, capped at university education if the highest reported level is ‘university or above’, to approximate the average years of education, weighted by the proportions completing each level. Similarly, if age groups are reported, we will calculate a weighted average age by assigning a midpoint to age groups other than the oldest category and a quarter of the range from the lower bound to the maximum reported age or to the life expectancy if no upper bound is reported for the oldest age group (e.g. 60 and above).

For reports involving participants in multiple strata, we will extract stratum-specific outcomes provided that each stratum has the required sample size of ≥ 50. Such reports will contribute more than one comparison to the dataset. If any stratum fails to achieve the required sample size, we will combine the participants and average the outcomes and cohort characteristics across the strata, weighted by stratum size or the inverse variance when extracting adjusted outcome measures. For instance, an article reports the numbers of HIV-seropositive and HIV-seronegative participants stratified by age group as follows: 22 and 21 for participants aged < 50 years (Stratum 1) and 25 and 26 for participants aged ≥ 50 years (Stratum 2). Since the size of Stratum 1 (*n* = 43) is less than 50, the combined data to extract will correspond to a single sample of 94 participants of mixed age groups, of whom 47 are HIV seropositive.

When there are multiple subgroups within an HIV serostatus group, we will extract data only from subgroups that are most similar to the other HIV serostatus group. For instance, if a study enrolled HIV-seropositive smokers and HIV-seronegative participants who did and did not smoke, only data from the HIV-seronegative smokers will be extracted for analysis. Subcohorts with nonoverlapping membership will be treated as independent observations. If unique membership between cohorts from the same study cannot be verified, either through the information provided in the text or from contacting the authors, the largest cohort will be selected for extraction.

We will employ two methods for extracting NCI outcomes. The first of these is direct extraction, which applies to articles reporting the frequency or proportions of NCI by serostatus group from which a measure of excess burden will be calculated. The second is indirect extraction and applies to articles reporting neuropsychological test scores or a summary test score. With indirect extraction, we will first convert the individual test *Z* scores to *T* scores (mean = 50, *SD* = 10) using the reported mean and standard deviation (SD), which will then be averaged over the individual neuropsychological tests and for each serostatus group. In the second step, these global *T* scores and the corresponding SDs will be used to construct a normal distribution of participant scores. We will then rescale this distribution to global deficit scores. NCI cases will be counts of participants with the global deficit score exceeding 0.5 by this diagnostic criterion [30]. When raw scores, rather than *Z* scores, are reported, we will standardize the test scores using HIV-seronegative participants as the normative population. If an article reports on more than one diagnostic instrument or criteria, we will extract only NCI outcomes from the neuropsychological test battery, as this is considered the diagnostic gold standard, or from the instrument and the diagnostic criteria that give the strongest evidence of a statistical difference. Only baseline NCI outcomes will be extracted from observational cohort studies.

We will contact study authors by email in three attempts over a 2-week period for clarifications on methodological aspects (e.g. study design, participant characteristics, methods to compute outcomes) that will allow us to better assess the eligibility of a report for inclusion or for missing statistics (e.g. unreported standard deviations) required for meta-analysis. In the event that the contacted authors provide an insufficient response, a consensus judgment will be exercised to determine eligibility or imputation of missing statistics will follow (see below).

### Assessment of risk of bias

Risk of bias will be assessed using an adapted Newcastle–Ottawa scale. This adapted version incorporates the design elements specific to cross-sectional studies [37] and includes modifications to refine question items for more relevance to HAND (Additional file 4). The scale comprises seven items organized into three bias domains (participant selection, comparability, and outcomes), with a maximum possible score of nine for studies with a very low risk of bias. Study quality will be divided into ‘good’ (low risk of bias: scores of 3–4 in selection, 1–2 in comparability, and 2–3 in outcomes), ‘fair’ (medium risk of bias: scores of 2 in selection, 1–2 in comparability, and 2–3 in outcomes), and ‘poor’ (high risk of bias: scores of < 2 in selection, 0 in comparability, or < 2 in outcomes). Two reviewers (G. H., G. M.) will independently conduct the quality assessment, with a third reviewer (A. R.) available to adjudicate in the event of any disagreements.

### Data synthesis

We will first describe the included reports and summarize key study-level characteristics. Next, we will pool the PRs from all eligible studies using the inverse-variance random-effects method of meta-analysis (DerSimonian-Laird) with the Knapp-Hartung adjustment for standard errors [38], stratifying the results by whether or not the studies utilized MoHE-adjusted controls. The Cochran’s *Q* and *I*^2^ statistics will be computed to assess the heterogeneity of the pooled PRs. An *I*^2^ value exceeding 50% is taken to indicate substantial heterogeneity [39]. Hypothesis testing of a difference in the pooled PRs from studies with MoHE-adjusted controls and MoHE-naive controls will be performed. We will assess the effect of influential studies by removing studies one at a time or in pairs, whereby one MoHE-adjusted study and one MoHE-naive study are removed simultaneously until all possible pairs are exhausted and re-pooling the PRs from the remaining study population.

We will assess small-study effects by evaluating the symmetry in the distribution of log PRs through visual inspection of a funnel plot and by statistical testing using the Egger’s test for MoHE-adjusted studies and MoHE-naive studies [40]. The Duval and Tweedie trim-and-fill technique will be used to adjust the pooled PRs in the presence of small-study effects (*P* ≤ 0.100) [41].

In accordance with the MOOSE guideline [28], exploratory analyses of subgroups and potential moderators will be performed and detailed as follows. We will perform subgroup analyses by study location (World Health Organization regions), age groups (mean age < 50 *vs*. ≥ 50 years), whether the study exclusively enrolled comorbid participants, outcome extraction (direct *vs*. indirect), NCI diagnostic criteria, and whether the PRs were adjusted for demographics (age, sex, education) and/or any comorbidities. For each level of a subgroup, we will compare the pooled PRs between MoHE-adjusted studies and MoHE-naive studies using the *Q* test for between-group differences. However, we acknowledge that these statistics may be inestimable or unreliably estimated in a number of subgroups due to sparse studies in either MoHE category.

Finally, we will conduct random-effects meta-regression to explore factors that may modify the pooled PRs. We will consider both continuous and ordinal study-level covariates, including mean age, nadir CD4 count, the proportion of HIV-seropositive participants on cART, number of assessed neurocognitive domains, and study-quality ranking. Because we anticipate fewer studies than would be typically required for multivariate analysis, the effects of these covariates on the pooled PRs will be assessed separately. Where possible, we will further classify MoHE-adjusted studies into behavioural exposure (e.g. men who have sex with men, people who use drugs or alcohol, high-risk heterosexuals) and nonbehavioural exposure (e.g. haemophiliac patients, people who were exposed to HIV perinatally) or similar distinctions to contrast the effects of different MoHEs on the excess burden of NCI. Missing covariate values will be imputed by the average value from the remaining studies sharing the same location and, for MoHE-adjusted studies, MoHE group.

## Discussion

This protocol outlines the methodology and analytical approaches for a forthcoming systematic review and meta-analysis of excess burden of NCI in PLWH on a global scale. Findings from this review will provide insights into the global burden of NCI while considering the psychosocial dimension of PLWH that the MoHE represents. By reviewing controlled studies, characterizing intergroup comparability, and employing a relative measure of NCI burden, we shift the focus from a unidimensional quantification of NCI burden to investigation of disparities in its distribution. A recent push to revise the nomenclature for HAND underscores a growing concern regarding the clinical utility of the prevailing approach in current diagnostics, which tends to result in excessive overdiagnoses of NCI cases [11]. The results of our meta-analysis will contribute to the current debate surrounding the revision of the HAND case definition, particularly on the credible attribution of NCI to HIV infection. We stress on the unique psychosocial circumstances of HIV key populations, who are the main target groups of public health interventions [42], as a potential moderating or competing factor for NCI in comparison to the conventional undestranding that ascribes NCIE solely to HIV-induced brain pathology.

In selecting the literature for inclusion, we will exclude publications without full text such as conference materials. This type of publications often has missing data and provides insufficient information for an adequate assessment of risk of bias. Additionally, we anticipate that the number of such publications will be low, and their exclusion will not impact the overall body of evidence. We have chosen not to impose language restrictions and instead plan to utilize publicly accessible online translation services for publications in languages other than English or Indonesian. While this may introduce inaccuracies during data extraction, it allows us to capture findings beyond those of leading research groups in high-income, English-speaking countries. We acknowledge that our requirement of ≥ 90% participants in both serostatus groups sharing an identical MoHE to be classified as MoHE-adjusted studies may be too restrictive and result in only a handful of reports meeting this criterion, which may limit our ability to investigate the full range of covariates in our planned subgroup and meta-regression analyses. Although somewhat arbitrary, the requirement of ≥ 90% participants having a uniform MoHE, while exceptionally high, is a conservative condition for attributing the NCI burden to a specific MoHE.

### Supplementary Information

**Additional file 1. **PRISMA-P 2015 Checklist.

**Additional file 2.** Search Terms.

**Additional file 3.** Data Extraction Form.

**Additional file 4.** The Modified Newcastle-Ottawa Scale for the Risk of Bias Assessment of Neurocognitive Impairments in People Living with HIV.

## Data Availability

Not applicable.

## Abbreviations

cART: Combination antiretroviral treatment
HAND: HIV-associated neurocognitive disorders
HIV: Human immunodeficiency virus
ISRCTN: International Standard Randomised Controlled Trial Number
MoHE: Mode of HIV exposure
MOOSE: Meta-analysis Of Observational Studies in Epidemiology
NCI: Neurocognitive impairment
OR: Odds ratio
PLWH: People living with HIV
PR: Prevalence ratio
PRISMA: Preferred Reporting Items for Systematic Reviews and Meta-Analyses
PRISMA-P: Preferred Reporting Items for Systematic Reviews and Meta-Analysis Protocols
PROSPERO: International Prospective Register of Systematic Reviews
SD: Standard deviation
