# Supplementary material for: Mode of HIV exposure and excess burden of neurocognitive impairment in people living with HIV: a protocol for systematic review and meta-analysis of controlled studies
Source: Syst Rev. 2023 Nov 16;12:214. doi: 10.1186/s13643-023-02371-6 (PMC10652586; doi:10.1186/s13643-023-02371-6)
Supplement: Supplementary file 2 — Additional file 2. Search Terms. [file 13643_2023_2371_MOESM2_ESM.docx]

**Search Terms**

| # | Searches |  |
| --- | --- | --- |
| 1. | exp HIV/ OR exp HIV-1 or exp HIV-2/ OR exp Acquired Immunodeficiency Syndrome/ |  |
|  | OR exp HIV Infections |  |
| 2. | (HIV or HIV infect* OR human immunodeficiency virus).tw. |  |
| 3. | (human ADJ (immunodefici* OR immuno-defici* OR immunedefici* OR immune-defici*) |  |
|  | ADJ virus).tw. |  |
| 4. | (acquired ADJ (immunodefici* OR immuno-defici* OR immunedefici* OR immune- |  |
|  | defici*) ADJ virus).tw. |  |
| 5. | 1 OR 2 OR 3 OR 4 |  |
| 6. | Neurocognitive Disorders/ OR Cognitive Dysfunction/ OR Cognition Disorders/ OR AIDS |  |
|  | Dementia Complex/ |  |
| 7. | (neurocogniti* OR neuro-cogniti* OR cogniti* OR neuropsychol* OR neuro-psychol* OR |  |
|  | neuropsychiat* OR neuro-psychiat*) ADJ (disorder$ OR impairment$ or deficit$ OR |  |
|  | deteriorati* OR degradati* OR degenerati*).tw. |  |
| 8. | 6 OR 7 |  |
| 9. | Control Groups/ |  |
| 10. | (control* OR comparison$).tw. |  |
| 11. | (HIV-uninfected OR HIV-noninfected OR HIV-seronegative OR HIV-negative OR |  |
|  | sero-negative OR seronegative OR sero-status or serostatus) |  |
| 12. | 9 OR 10 OR 11 |  |
| 13. | 5 AND 8 AND 12 |  |
| 14. | (animal$ NOT human$).sh,hw. |  |
| 15. | 13 NOT 14 |  |
| 16, | limit 15 to dd=20070101-20230630 |  |
| 17. | limit 15 to rd=20070101-20230630 |  |
| 18. | limit 15 to ep=20070101-20230630 |  |
| 19. | 16 OR 17 OR 18 |  |
