# Supplementary material for: Mode of HIV exposure and excess burden of neurocognitive impairment in people living with HIV: a protocol for systematic review and meta-analysis of controlled studies
Source: Syst Rev. 2023 Nov 16;12:214. doi: 10.1186/s13643-023-02371-6 (PMC10652586; doi:10.1186/s13643-023-02371-6)
Supplement: Supplementary file 3 — Additional file 3. Data Extraction Form. [file 13643_2023_2371_MOESM3_ESM.docx]

**Data Extraction Form**

**Title :** Mode of HIV Exposure and Excess Burden of Neurocognitive Impairment in People Living with HIV: A Protocol for Systematic Review and Meta-analysis of Controlled Studies

**Registration No :** CRD42021271358

1. **General Information**

| Date form completed (dd/mm/yyyy) | /  / |
| --- | --- |
| Name of person extracting data |  |
| Study ID |  |
| Study title |  |
| Year of publication |  |
| Country in which the study conducted |  |
| First author |  |

1. **Population**

| Type of control – 1 (restrictive definition of MoHE) | Type of control group used:  Generic (control group consists of HIV-negative participants and no description of the derivation from the same population-at-risk as HIV-positive participants).  MoHE (control group consists of at least 90% participants with same risk as HIV-positive participants). |
| --- | --- |
|  | Type of HIV risk group used:  Not described (generic).  MSM: men who have sex with men.  PWUDA: people who use drugs and/or alcohol.  High-risk heterosexuals: examples include female sex workers or other non-homosexuals with multiple partners.  HIV perinatal: those vertically infected during pregnancy, at birth or postpartum.  Other: … |
| Type of control – 2 (liberal definition of MoHE) | Type of control group used:  Generic (control group consists of HIV-negative participants and no description of the derivation from the same population-at-risk as HIV-positive participants).  MoHE (control group consists of at least 50% participants with same risk as HIV-positive participants or difference across population-at-risk groups was controlled with matching or regression analysis). |
|  | Type of HIV risk group used:  Not described (generic).  MSM: men who have sex with men.  PWUDA: people who use drugs and/or alcohol  High-risk heterosexuals: examples include female sex workers or other non-homosexuals with multiple partners  HIV perinatal: those vertically infected during pregnancy, at birth or postpartum  Other: … |
| Comorbid condition applicable to all |  |

1. **Methods**

| Diagnostic instrument | NP Battery  MMSE  CogState  MoCA  IHDS  Other: … |
| --- | --- |
| Reference population | Norms  Control  Cut-off (default value of instrument according to manual/literature) |
| Correction for norms | Does calculation of neurocognitive perfomance based on adjusted normative value of sample demographics (gender, age, education, etc)?  Yes (normative value adjusted to demographics using regression or stratification of demographics variable)  No (no description available) |
| Number of neurocognitive domains evaluated |  |
| Diagnostic criteria | Frascati  Global Deficit Scale (GDS)  International HIV Dementia Scale (IHDS)  Multivariate normal comparison  Other screening tools with certain cut-off value are reported as it is or based on assumption |
| Extraction method | Direct: number of cases stated in the article  Indirect: the article reported global score in a standard scale with standard cut-off value for neurocognitive impairment diagnosis |

1. **Participants**

| Number of participants based on serostatus | HIV-positive | HIV-negative |
| --- | --- | --- |
| Number of participants with neurocognitive impairment based on serostatus | HIV-positive | HIV-negative |
| Mean (or median) nadir CD4 value | for HIV-positive only cases in the study | |
| Proportion of HIV-positive cases on antiretroviral therapy (during the study) |  | |
| Mean (or median) age value based on serostatus | HIV-positive | HIV-negative |
| Proportion of sex (female) based on serostatus | HIV-positive | HIV-negative |
| Proportion of sex (male) based on serostatus | HIV-positive | HIV-negative |
| Mean (or median) duration of education based on serostatus | HIV-positive | HIV-negative |
| Proportion of never or not smoke tobacco based on serostatus | HIV-positive | HIV-negative |
| Design/analytic adjustment | Is there any adjustment with matching or regression analysis for demographic characteristics (age)?  Yes  No | |
|  | Is there any adjustment with matching or regression analysis for demographic characteristics (sex)?  Yes  No | |
|  | Is there any adjustment with matching or regression analysis for demographic characteristics (education)?  Yes  No | |
|  | Is there any adjustment with matching or regression analysis for comorbidities characteristics (depression)?  Yes  No | |
|  | Is there any adjustment with matching or regression analysis for demographic characteristics (other)?  Yes  No | |
| List of other comorbidities (if any) |  | |

1. **Outcomes**

| Pooled effect size from n  (RR, OR, etc) |  |
| --- | --- |
| Relative Effect Size (RES) from regression |  |
| Final pooled effect |  |

1. **Remarks (if any)**

| Comments |  |
| --- | --- |
