# Supplementary material for: Mode of HIV exposure and excess burden of neurocognitive impairment in people living with HIV: a protocol for systematic review and meta-analysis of controlled studies
Source: Syst Rev. 2023 Nov 16;12:214. doi: 10.1186/s13643-023-02371-6 (PMC10652586; doi:10.1186/s13643-023-02371-6)
Supplement: Supplementary file 4 — Additional file 4. The Modified Newcastle-Ottawa Scale for the Risk of Bias Assessment of Neurocognitive Impairments in People Living with HIV. [file 13643_2023_2371_MOESM4_ESM.docx]

The Modified Newcastle-Ottawa Scale for the Risk of Bias Assessment of Neurocognitive Impairments in People Living with HIV

Adapted from Herzog et al (2013),^1^ the scale comprises 3 domains of risk of bias in **selection** (4 items, max. 4 stars), **comparability** (1 item, max. 2 stars), and **outcomes** (2 items, max. 3 stars) for a maximum total score of 9 stars (very low risk of bias).

| **Selection** | | |
| --- | --- | --- |
| 1. Representativeness of the sample | | |
| ☆ | | Truly representative of the average in the target population (all subjects or random sampling) *OR* somewhat representative of the average in the target group (non-random sampling). |
| 2. Sample size | | |
| ☆ | | Justified with sample size calculation and satisfactorily achieving the target enrolmnt *OR* having a sufficiently large number of participants (*n*>300) in both serostatus groups. |
| 3. Non respondents | | |
| ☆ | | Comparability of characteristics between enrolled subjects and those excluded for reason other than ineligibility is established. |
| 4. Ascertainment of the exposure | | |
| ☆ | | All HIV-infected participants were diagnosed with HIV *AND* all HIV-uninfected participants were tested negative for HIV within 12 months prior to the neuropsychological assessment. |
|  | | |
| **Comparability** | | |
| 5. Comparability | | |
| ☆☆ | | Comparability of characteristics between HIV-seropositive participants and HIV-seronegative participants with regards to demographics (age, sex, education) *AND* comorbidities. |
| ☆ | | Comparability of characteristics between HIV-seropositive participants and HIV-seronegative participants with regards to (age, sex, education) *OR* comorbidities. |
|  | | |
| **Outcomes** | | |
| 6. Assessment of outcomes | | |
| ☆☆ | | Diagnosis of neurocognitive impairment on the basis of a neuropsychological test battery assessing at least 5 of the ability domains described in Antinori et al (2007).^2,†^ |
| ☆ | | Diagnosis of neurocognitive impairment on the basis of a neuropsychological test battery assessing 2-4 of the ability domains described in Antinori et al (2007)^2,†^ *OR* use of screening analogues (e.g., IHDS MMSE, MoCA). |
| 7. Statistical test | | |
| ☆ | | Statistical test used to analyze the data clearly described, appropriate and measures of association presented including the absolute or relative difference by HIV serostatus with confidence intervals or the probability level (*P*-value). |
| IHDS: International HIV Dementia Scale; MMSE: Mini Mental State Examination; MoCA: Montreal Cognitive Assessment. | | |
| ^†^: | These domains are abstraction-executive functioning; attention-information processing; complex perceptual motor skills; language; memory (including learning and recall), sensory perceptual abilities *or* simple motor skills. | |

1. Herzog R, Álvarez-Pasquin MJ, Díaz C, Del Barrio JL, Estrada JM, Gil Á. Are healthcare workers’ intentions to vaccinate related to their knowledge, beliefs and attitudes? A systematic review. BMC Public Health. 2013. https://doi.org/10.1186/1471-2458-13-154.

2. Antinori A, Arendt G, Becker JT, Brew BJ, Byrd DA, Cherner M, et al. Updated research nosology for HIV-associated neurocognitive disorders. Neurology. 2007. https://doi.org/10.1212/01.WNL.0000287431.88658.8b.
